# Chemical-induced colitis lowers mitochondrial bioenergetic function in the colonic epithelium with minimal impacts on the proteome

**DOI:** 10.21203/rs.3.rs-7697508/v1

**Published:** 2025-10-30

**Authors:** McLane M. Montgomery, Masara A. Al Obaidi, Raphael T. Aruleba, Polina Krassovskaia, Emely A. Pacheco, Edziu Franczak, Brett R. Chrest, Thomas D. Green, Tonya N. Zeczycki, Joeseph M. McClung, Kelsey H. Fisher-Wellman

**Affiliations:** East Carolina University; East Carolina University; Wake Forest University; Wake Forest University; Wake Forest University; Wake Forest University; Wake Forest University; Wake Forest University; East Carolina University; Wake Forest University; Wake Forest University

**Keywords:** DSS, mitochondrial respiration, epithelial barrier, energy transduction, inflammation

## Abstract

Dextran Sulfate Sodium (DSS) is widely used to model colitis due to its ability to disrupt the colonic epithelial barrier and trigger inflammation. While DSS is a valuable tool for studying colitis-related diseases, its impact on mitochondrial bioenergetics and the proteomic landscape of colonic tissue remains poorly understood. To address this gap, we administered 3% DSS in drinking water to C57BL/6J mice and analyzed resected colonic tissue from treated and control mice. Longitudinally opened colon segments were cleaned and subjected to high-resolution respirometry and mass spectrometry-based proteomic profiling. DSS treatment led to a global lowering of mitochondrial respiration, with the most pronounced impairments observed in complex I-supported respiration. Proteomic analysis revealed that these functional deficits occurred largely independently of changes in the mitochondrial proteome, except for an apparent upregulation of NIPSNAP1, a mitophagy-related protein. However, lentiviral knockdown of NIPSNAP1 in HCT116 cells did not rescue the observed bioenergetic defects, suggesting it is not the primary driver. Collectively, our findings show that DSS impairs mitochondrial respiration in the colon—most notably at complex I—without major alterations to the mitochondrial proteome. Given the role of mitochondrial dysfunction in various diseases, these effects should be carefully considered when using DSS-based models to study colitis pathophysiology.

## INTRODUCTION

Dextran Sulfate Sodium (DSS) is widely used to induce colitis in animal models by disrupting the intestinal epithelial barrier and triggering inflammation. The DSS model effectively recapitulates key features of human inflammatory bowel disease (IBD), providing a controllable system to study inflammation-associated pathologies^1–5^. Maintaining the integrity of the intestinal barrier is an energy-intensive process, primarily supported by mitochondrial oxidative phosphorylation (OxPhos)^6^. Disruption of mitochondrial ATP production has been shown to impair barrier function and increase intestinal permeability^7^. Conversely, mice with genetic variants that enhance mitochondrial oxidative capacity are protected from experimentally induced colitis^8^, underscoring the central role of mitochondrial energetics in maintaining intestinal homeostasis.

Mitochondria, present in nearly all cell types except red blood cells, are essential for ATP synthesis, metabolic regulation, and cell fate decisions^9–10^. In intestinal epithelial cells (IECs), mitochondrial function is highly dynamic and influenced by factors such as cell maturation, location within the crypt-villus axis, nutrient availability, and disease state. Mitochondrial alterations have been implicated in a range of pathologies including IBD and colorectal cancer^10–13^. In ulcerative colitis, colonocytes exhibit altered nutrient metabolism, with decreased butyrate oxidation and increased reliance on glucose and glutamine^14^. Furthermore, mitochondrial dysfunction contributes to elevated reactive oxygen species (ROS) production, promoting inflammation, apoptosis, and DNA damage^15–17^. Understanding how DSS-induced colitis alters mitochondrial function in colonic tissue is therefore essential for elucidating its physiological impact and relevance to human disease.

To address this, we evaluated how DSS-induced colitis alters mitochondrial energy metabolism and proteomic composition within the colon. Colon strips from control and DSS-treated C57BL/6J mice were chemically and mechanically permeabilized to assess mitochondrial OxPhos function and proteome composition. Together, these experiments provided a comprehensive assessment of how DSS-induced colitis impacts mitochondrial function and composition, highlighting intrinsic alterations in OxPhos capacity and proteome expression in response to chronic colonic inflammation.

## RESULTS

DSS-induced colitis reduces respiratory complex I function despite minimal changes to the proteome.

**Figure 1:**
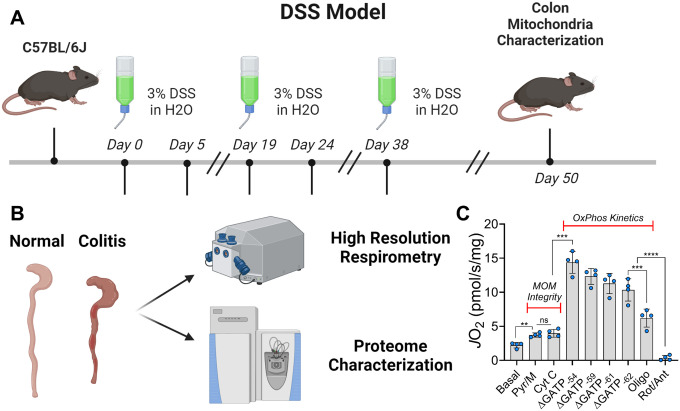
Multi-platform workflow for assessing mitochondrial respiration and composition in DSS-treated colon tissue. **A)** Graphical representation of the timeline of DSS treatment via drinking water. **B)** Schematic depicting resection of normal and DSS-treated colon and the respirometry and mass-spectrometry based assays conducted. **C)** Optimization of the creatine kinase energetic clamp technique on mechanically and chemically permeabilized colon strips. N=4 independent experiments. Data presented as mean ± SEM and analyzed by unpaired t-test *P<0.05, **P<0.01, ***P<0.001, ****P<0.0001. CK (20 U/mL), ATP (5 mM), PCR (5 mM, 14 mM, 20 mM), pyruvate (Pyr; 5 mM), malate (mal; 1 mM), cytochrome C (Cyt C, 10 μM), oligomycin (Oligo, 0.02 μM), rotenone (Rot, 0.5 μM), antimycin A (Ant A, 0.5 μM).

**Figure 2:**
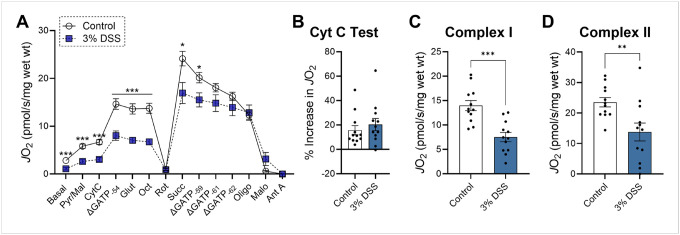
Lower mitochondrial respiration is a key characteristic of colitis afficted colon tissue. All assays were performed on mechanically and chemically (saponin) permeabilized colon strips from both control and DSS-treated mice. **A)** Creatine kinase energetic clamp (OxPhos kinetics) respirometry technique. **B)** Mitochondrial outer membrane integrity cytochrome C test. **C)** Mitochondrial complex I-specific respiration (stimulated by pyruvate/malate, ΔGATP = −54 kJ/mol). **D)**Mitochondrial complex II-specific respiration; N=12 independent experiments. Data are presented as mean ± SEM and analyzed by unpaired t-test. *P<0.05, **P<0.01, ***P<0.001. Substrates and inhibitors utilized are indicated as follows: CK (20 U/mL), ATP (5 mM), PCR (5 mM, 14 mM, 20 mM), pyruvate (Pyr; 5 mM), malate (mal; 1 mM), glutamate (glut; 5 mM), octanoyl-carnitine (oct; 0.2 mM), succinate (Succ; 5 mM) cytochrome C (Cyt C, 10 μM), oligomycin (Oligo, 0.02 μM), rotenone (Rot, 0.5 μM), malonate (Malo, 20mM), antimycin A (Ant A, 0.5 μM).

To investigate how colitis impacts mitochondrial bioenergetics, we initiated treatment in mice with 3% DSS (Fig. 1A), followed by assessment of mitochondrial respiratory flux and proteome composition in resected colonic tissue from both control and DSS-treated animals (Fig. 1B). Prior to functional analysis, colon strips were chemically (saponin) and mechanically permeabilized to allow direct access to the mitochondrial network. In mammalian cells, the majority of the adenylate pool is maintained as ATP, with ATP free energy of hydrolysis values (ΔGATP) typically ranging from − 56 to −64 kJ/mol^18^. To assess OxPhos kinetics across a physiological range of ATP resynthesis demands, we employed the creatine kinase (CK) energetic clamp in permeabilized colon tissue (Fig. 1C)^19^. Upon stimulation with saturating concentrations of carbon substrates, DSS-treated colon strips exhibited a widespread lowering of mitochondrial respiratory flux (*J*O_2_) (Fig. 2A). To confirm that these changes were not due to mitochondrial outer membrane damage, we performed a cytochrome c integrity test and observed no difference between treatment groups (Fig. 2B). Respiration driven by individual complexes revealed that the most pronounced decline occurred with complex I substrates at ΔGATP = −54 kJ/mol (Fig. 2C). Although complex II-supported respiration was also lower, the decrease was less substantial (Fig. 2D). The ratio of complex I- to complex II-supported respiration was lower in DSS-treated mice. Together, these results demonstrate that DSS-induced colitis is characterized by a marked suppression of mitochondrial respiration, with a preferential deficit in complex I-driven bioenergetics.

**Figure 3:**
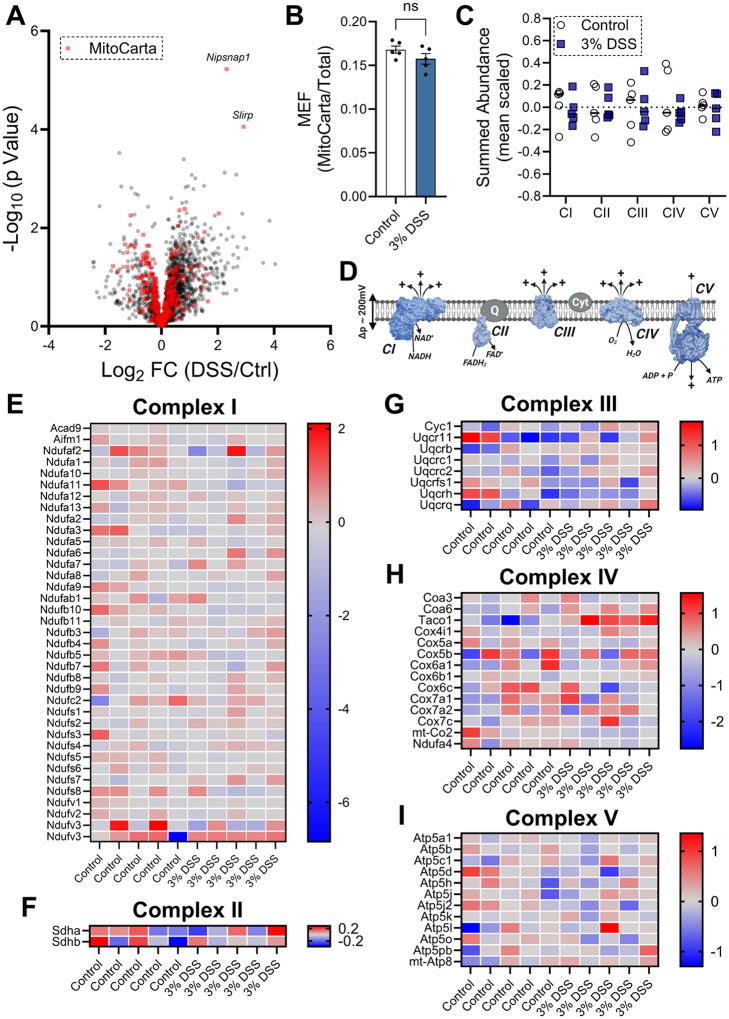
The mitochondrial proteome is relatively unchanged by chemically-inducedcolitis. **A)** Volcano plot comparing the mitochondrial to non-mitochondrial proteomes in resected colon tissue (mitochondrial proteins depicted in red). **B)** Ratio of mitochondrial to non-mitochondrial proteins (MEF) detected through mass-spectrometry analysis. **C)** Summed abundance of detected proteins categorized to each specific complex. **D)** Graphical representation of the mitochondrial electron transport and oxidative phosphorylation systems. **E)** Heat-map depicting the expression of proteins involved in complex I function. **F)**Heat-map depicting the expression of proteins involved in complex II function. **G)** Heat-map depicting the expression of proteins involved in complex III function. **H)** Heat-map depicting the expression of proteins involved in complex IV function. **I)** Heat-map depicting the expression of proteins involved in complex V function; N=5. Data are presented as mean ± SEM and analyzed by unpaired t-test (Fig 3B) and multiple unpaired t-test (Fig 3C). *P<0.1.

Given the observed alterations in mitochondrial bioenergetics following DSS treatment, we next sought to determine whether these functional changes were associated with alterations in the proteome. To this end, we performed quantitative proteomic profiling of control and DSS-treated colon tissue using an nLC-MS/MS-based approach. Importantly, colon strips were analyzed without prior permeabilization, enabling comprehensive assessment of both mitochondrial and non-mitochondrial proteins. Proteomic analysis revealed that the overall composition of the colon tissue proteome remained largely unchanged following DSS treatment. Notably, the only significantly altered proteins were NIPSNAP1, associated with mitophagy signaling, and SLIRP, involved in mitochondrial mRNA stabilization (Fig. 3A). To assess relative mitochondrial content, we calculated the mitochondrial enrichment factor (MEF), defined as the ratio of mitochondrial to non-mitochondrial protein abundance^20^. MEF remained unchanged between control and DSS-treated groups, indicating no global differences in mitochondrial protein abundance (Fig. 3B). The OxPhos proteome, comprising complexes I–V, was further analyzed at both the complex and individual protein levels. No significant differences were observed in the abundance of any OxPhos complex (Figs. 3C & 3D). At the individual protein level, only minor changes were detected within complexes I and II (Figs. 3E & 3F), and no notable changes were observed in complexes III, IV, or V (Figs. 3G–I). Together, these findings demonstrate that the bioenergetic alterations induced by DSS treatment occur independently of major changes in mitochondrial proteome composition.

NIPSNAP1 knockdown fails to rescue colitis-induced alterations in mitochondrial bioenergetic function.

**Figure 4:**
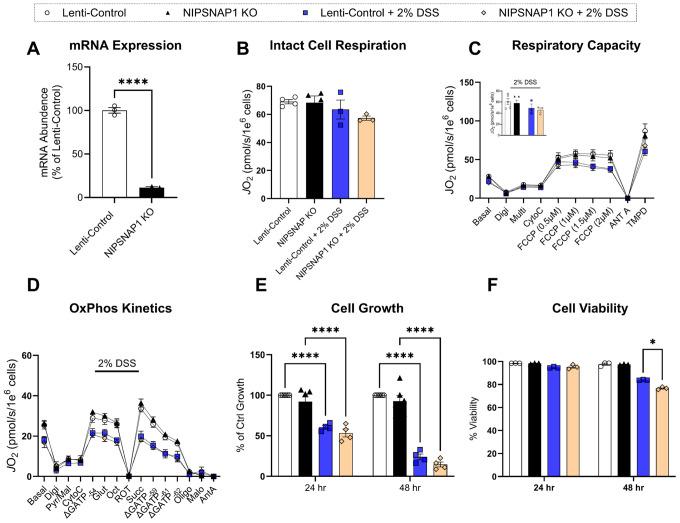
NIPSNAP1 KO does not alter mitochondrial bioenergetic function. All respirometry assays were performed in intact or chemically (digitonin) permeabilized HCT116 cells. **A)** mRNA abundance of NIPSNAP1 in HCT116 cells, displayed as percentage of control mRNA abundance **B)** Maximum intact cellular respiration. **C)** Maximal respiratory capacity in permeabilized cells including an extrapolated bar graph depicting changes in maximal respiration are due entirely to DSS treatment. **D)** Creatine kinase energetic clamp (OxPhos kinetics) respirometry technique in permeabilized cells. **E & F)** Cell growth and viability measured by Trypan Blue counting. N=3–5. Data are presented as mean ± SEM and analyzed by Two-way or three-way ANOVA. *P<0.05. **P<0.01, ***P<0.001, ****P<0.0001. Substrates and inhibitors utilized are indicated as follows: CK (20 U/mL), ATP (5 mM), PCR (5 mM, 14 mM, 20 mM), pyruvate (Pyr; 5 mM), malate (mal; 1 mM), glutamate (glut; 5 mM), octanoyl-carnitine (oct; 0.2 mM), succinate (Succ; 5 mM) cytochrome C (Cyt C, 10 μM), oligomycin (Oligo, 0.02 μM), rotenone (Rot, 0.5 μM), malonate (Malo, 20mM), antimycin A (Ant A, 0.5 μM),

Proteomic analysis of DSS-treated colon tissue identified NIPSNAP1 as the most highly upregulated protein based on an adjusted p-value of < 0.1 (Fig. 3A). To investigate its potential role in altering mitochondrial bioenergetic function, we used lentiviral particles targeting the 3’ UTR of NIPSNAP1 to generate knockdown (KO) HCT116 cells. These cells were subjected to assays assessing mitochondrial bioenergetics, cell growth, and viability, both in the presence and absence of 2% DSS to mimic colitis-like conditions. Knockdown efficiency was confirmed by qPCR (Fig. 4A). We first assessed intact cell respiration and found no difference in maximal respiration between any treatment group (Fig. 4B). To further probe mitochondrial function, cells were permeabilized with digitonin (15 μg/mL) and supplied with saturating carbon substrates. FCCP was then added to uncouple the electron transport system (ETS) from ATP synthesis. Under these conditions, maximal ETS capacity was unaffected by NIPSNAP1 knockdown (Fig. 4C). Additionally, when subjected to the CK clamp—used previously in colon tissue—NIPSNAP1 knockdown conferred no change in OxPhos capacity (Fig. 4D). DSS treatment was sufficient to lower both maximal respiration and OxPhos capacity, recapitulating the mitochondrial alterations observed *in vivo* (main effect 2% DSS, P < 0.01). However, lower OxPhos flux was independent of NIPSNAP1 knockdown, as no differences were observed between KO and control cells in either DSS-treated or untreated conditions. Cell growth was unaffected by NIPSNAP1 depletion and was reduced only in response to DSS (Figs. 4E). NIPSNAP1 knockdown reduced cell viability after 48 hours (Fig. 4F). Together, these findings demonstrate that DSS impairs OxPhos and cell proliferation in HCT116 cells, but that NIPSNAP1 upregulation is not required for these effects.

## DISCUSSION

The use of DSS to experimentally induce colitis remains a foundational tool for studying the mechanisms underlying colitis and its contribution to diseases such as IBD and colorectal cancer. In our study, DSS treatment resulted in a global lowering of mitochondrial respiratory flux, with the most substantial decreases observed at respiratory complex I. These findings align with previous work by Shearer et al., who reported reductions in both complex I and II activity in intestinal epithelial cells following DSS exposure. Notably, these DSS-treated IECs also exhibited impaired beta-oxidation, as evidenced by reduced respiration supported by butyrate—a key energy substrate preferentially utilized by healthy IECs. Although a compensatory shift toward glucose and glutamine metabolism was observed, overall OxPhos remained diminished^21^. Together, these data suggest that mitochondrial alterations in colitis are largely independent of carbon source availability and may contribute to the loss of epithelial barrier integrity, a hallmark of IBD^22–23^.

Our proteomic analyses revealed that lower OxPhos flux occurred without widespread changes in the mitochondrial proteome, with the exception of elevated expression of NIPSNAP1 and SLIRP. This suggests that DSS-induced mitochondrial alterations may not stem from altered gene expression but could instead result from post-translational modifications or disruptions in the structural integrity of mitochondrial protein complexes. Given NIPSNAP1’s established role as a mitophagy-related “eat-me” signal, we hypothesized that increased mitochondrial turnover might underlie the observed alterations^24–25^. However, lentiviral knockdown of NIPSNAP1 in HCT116 cells failed to impact mitochondrial respiration or cell growth and minimally reduced cell viability, indicating that its upregulation is not directly responsible for the bioenergetic deficits observed. These findings point to a more complex regulatory mechanism governing mitochondrial impairment in colitis that warrants further investigation.

Chronic colitis has been shown to cause extensive epithelial damage, promote expression of pro-inflammatory cytokines like TNF, and increase epithelial cell turnover^26–29^. These conditions can facilitate the accumulation of mitochondrial DNA (mtDNA) mutations, a phenomenon that has been repeatedly associated with increased susceptibility to tumorigenesis^30–32^. Indeed, large-scale sequencing studies have identified colorectal cancer as one of a subset of cancer types enriched for somatic mtDNA mutations^30^. Additionally, chronic colitis can promote the development of dysplastic lesions in the colon, further predisposing the tissue to malignant transformation^33–34^. In IECs, DSS treatment not only impairs mitochondrial energy metabolism but also increases oxidative stress and alters nutrient utilization—all of which are conditions known to drive tumor-promoting pathways^21,35^. These findings underscore the growing recognition of mitochondrial alterations as a key player in linking chronic colitis to colorectal cancer development.

One particularly intriguing finding was the DSS-induced upregulation of SLIRP, a protein known to bind the stem-loop structure of mitochondrial mRNAs, stabilizing transcripts and supporting translation^36–37^. While this function is typically protective, it may become maladaptive if SLIRP is stabilizing transcripts derived from damaged or mutated mtDNA, thereby propagating dysfunctional proteins within the OxPhos system. Collectively, our findings emphasize the need to carefully consider mitochondrial bioenergetics when using DSS-induced colitis models and provide important insight into early mitochondrial alterations that may contribute to inflammation associated colon pathologies (e.g., colorectal cancer). Further work is required to define the precise molecular events linking inflammation to persistent mitochondrial alteration and disease progression.

## METHODS

All procedures on experimental animals were approved by the Wake Forest University Animal Care and Use Committee (Animal Use Protocol Number A24–099) along with all relevant guidelines and regulations. Additionally, all experimental animal procedures were conducted in accordance with the ARRIVE 2.0 guidelines. All animal experiments were conducted at Wake Forest University in Winston Salem, NC. Mice were anesthetized using isoflurane. Before recovery from anesthetization, mice were euthanized via severing of the diaphragm and excision of the heart.

### Induction of colitis and colon resection

Healthy C57BL/6J mice obtained from Jackson Laboratories (JAX:000664) were utilized for all experiments. To induce colitis, mice were treated with 3% DSS at 16-weeks of age (Thermo Scientific; #J14489–22) as shown in Fig. 1A. Mice were administered DSS via drinking water in three separate five-day rounds (15 days). Each round of DSS treatment was separated by two consecutive weeks, in which mice were returned to normal drinking water. Water consumption and mouse weights were monitored throughout the entire 50-day treatment period. Control mice were kept on normal drinking water exclusively. After completion of DSS treatment, colons were resected from both control and DSS-treated mice by cutting the colon from the cecal valve to the anus. Resected colons were thoroughly rinsed in 1x PBS to remove all fecal material and cut open longitudinally to expose the intestinal lumen. Then, 1–2cm sections of colon were removed to be used in all proteomic and respiratory flux analyses.

### Respiratory flux analysis in permeabilized colon

High-resolution respirometry measurements were performed using the Oroboros Oxgraph-2k (O2k; Oroboros Instruments, Innsbruck, Austria) in a 1.0 mL reaction volume at 37°C as previously described^19^. For normalization to total protein, prior to each experiment the collected tissue samples were assessed for wet weight (mg). Tissue samples were permeabilized mechanically and with saponin (30μg/mL) and respiratory flux was measured using the creatine kinase (CK) technique. Within the CK clamp assay, the free energy of ATP hydrolysis (ΔG_ATP_) is calculated using the equilibrium constant for the CK reaction (*K’*_*CK*_) and is based upon the addition of known concentrations of creatine (CR), phosphocreatine (PCR), and ATP in the presence of excess amounts of CK. Calculation of ΔG_ATP_ at defined PCR concentrations was done as previously described^18^. For each CK clamp assay, saturating amounts of carbon substrates and inhibitors were employed. Substrates and inhibitors utilized are indicated as follows: CK (20 U/mL), ATP (5 mM), PCR (5 mM, 14 mM, 20 mM), pyruvate (Pyr; 5 mM), malate (mal; 1 mM), glutamate (glut; 5 mM), octanoyl-carnitine (oct; 0.2 mM), succinate (Succ; 5 mM) cytochrome C (Cyt C, 10 μM), oligomycin (Oligo, 0.02 μM), rotenone (Rot, 0.5 μM), malonate (Malo, 20mM), antimycin A (Ant A, 0.5 μM),

### Colon tissue lysis and protein digestion for nLC-MS/MS

Colon tissue from healthy and DSS-treated mice was homogenized and lysed in ice-cold 8 M Urea Lysis Buffer (8 M urea in 50 mM Tris, pH 8.0, 40 mM NaCl, 2 mM CaCl_2_, 1x cOmplete ULTRA mini EDTA-free protease inhibitor tablet), as described previously^38^. The samples were frozen on dry ice and thawed for three freeze-thaw cycles and further disrupted by sonication with a probe sonicator in three 5 s bursts set at an amplitude of 30 (Q Sonica, Newtown, CT). Samples were centrifuged at 10,000 × g for 10 min at 4°C to pellet insoluble material. Protein concentration was determined by BCA, and equal amounts of protein (200 μg, adjusted to 2.5 mg/mL with Urea Lysis Buffer) were reduced with 5 mM DTT at 32°C for 30 min, cooled to room temperature, and then alkylated with 15 mM iodoacetamide for 30 min in the dark. Unreacted iodoacetamide was quenched by the addition of DTT up to 15 mM. Initial digestion was performed with Lys C (Thermo Fisher) 1:100 w-w; 2 μg enzyme per 200 μg protein for 4 hr at 32°C. Following dilution to 1.5 M urea with 50 mM Tris (pH 8.0), 30 mM NaCl, 5 mM CaCl_2_, the samples were digested overnight with trypsin (Promega, Madison, WI) 50:1 w/w, protein:enzyme at 32°C. Samples were acidified with 0.5% TFA and centrifuged at 10,000 × g for 10 min at 4°C to pellet insoluble material. Supernatant containing soluble peptides was desalted on a 50 mg tC18 SEP-PAK solid phase extraction column (Waters, Milford, MA) and eluted (500 μL 25% acetonitrile/0.1% TFA and 2 × 500 μL 50% acetonitrile/0.1% TFA). The 1.5 mL eluate was frozen and lyophilized.

### nLC-MS/MS

As previously described^38^, peptide fractions were suspended in 0.1% formic acid at a concentration of 0.25 μg/μL, following peptide quantification (ThermoFisher). All samples were subjected to nanoLC-MS/MS analysis using an UltiMate 3000 RSLCnano system (Thermo Fisher) coupled to a Q Exactive PlusHybrid Quadrupole-Orbitrap mass spectrometer (Thermo Fisher) via nanoelectrospray ionization source. For each injection of 4 μL (1 μg), the sample was first trapped on an Acclaim PepMap 100 20 mm × 0.075 mm trapping column (Thermo Fisher) 5 μl/min at 98/2 v/v water/acetonitrile with 0.1% formic acid, after which the analytical separation was performed over a 90-min gradient (flow rate of 300 nanoliters/min) of 3 to 30% acetonitrile using a 2 μm EASY-Spray PepMap RSLC C18 75 μm × 250 mm column (Thermo Fisher) with a column temperature of 55°C. MS1 was performed at 70,000 resolution, with an AGC target of 1 × 10^6^ ions and a maximum IT of 60 ms. MS2 spectra were collected by data-dependent acquisition (DDA) of the top 20 most abundant precursor ions with a charge greater than 1 per MS1 scan, with dynamic exclusion enabled for 30s. Precursor ions were filtered with a 1.0 m/z isolation window and fragmented with a normalized collision energy of 30. MS2 scans were performed at 17,500 resolution, AGC target of 1 × 10^5^ ions, and a maximum IT of 60 ms.

### Data analysis for label-free proteomics

Proteome Discoverer 2.2 (PDv2.2) was used for raw data analysis. Default search parameters included oxidation as a variable modification (15.995 Da on M) and carbamidomethyl (57.021 Da on C) as a fixed modification. Data were searched against both the Uniprot Mus musculus reference proteome and mouse Mito Carta 3.0 database^39^. As previously described^20^, PSMs were filtered to a 1% FDR and grouping to unique peptides was also maintained at a 1% FDR at the peptide level. Strict parsimony was used to group peptides to proteins, and proteins were again filtered to 1% FDR. MS1 precursor intensity was used for peptide quantification, and low abundance resampling was used for imputation. High confidence master proteins were used to determine mitochondrial enrichment factor (MEF) by quantifying the ratio of mitochondrial protein abundance (identified using the MitoCarta 3.0 database) to total protein abundance^20^. Sample specific MEF was then used to first normalize all proteomics data to total mitochondrial protein abundance. Further normalization was performed by converting individual abundances of each quantified mitochondrial protein to percent contribution to total mitochondrial abundance. For quantification of the OxPhos complexes, we summed the percent contribution of all subunits quantified that match to MitoCarta 3.0 annotated subunits of CI, CII, CIII, CIV, and CV.

### Culturing of CRC cells

HCT116 cells were cultured in RPMI-1640 (Thermo Scientific; 61870–036) supplemented with 10% FBS and 1% penicillin/streptomycin and incubated at 37°C in 5% CO2 as previously described^42^. For respirometry experiments and cell maintenance, cells were split or utilized once they reached 70–80% confluence. Fresh media was supplied to the cells every 48 hours. For DSS treatment experiments, cells were cultured in RPMI-1640 media supplemented with 2% DSS (w/v) or normal RPMI as a control. Cells were cultured for 24 hours in DSS supplemented media for all respirometry assays and for 24–48 hours for all cell growth and viability assays.

### Lentiviral NIPSNAP1 Knockdown in CRC cells

HCT116 cells were cultured in RPMI-1640 supplemented with 10% FBS and 1% penicillin/streptomycin and incubated at 37°C in 5% CO2. Human shRNA lentiviral particles targeting the 3’UTR region of NIPSNAP1 (3’ UTR: 5’GCTGACAAGTTCTGAGGATTA3) and one scramble shRNA control (0.5mL each > 10^9^TU/mL) (Addgene; #1864) were generated. To facilitate effectiveness of the introduced lentiviral particles, Polybrene (Santa Cruz Biotechnology; sc-134220) was added at 5μg/mL to the treated cells as previously described^42^. Cells were cultured in both Polybrene and the NIPSNAP1/control virus for 72 hours. After infection, both were removed. Cells were then given fresh media and subjected to puromycin selection by continuous exposure to puromycin (2μg/mL) in the culture media. Cultured cells were then maintained at a constant puromycin concentration of 1μg/mL.

### Quantification of mRNA Expresion

To confirm NIPSNAP1 knockdown, RNA was isolated and purified from NIPSNAP1 KO HCT116 cells using the PureLink RNA Mini Kit (Thermo Scientific; 12183018A). As previously described^42^, RNA was then converted to cDNA for qPCR analysis using the SuperScript IV reverse transcriptase (Invitrogen; LT-02241). Primers for the gene of interest (NIPSNAP1) and endogenous control (18S rRNA) were purchased from Thermo Fisher Scientific (Hs00186287, #HS99999901_s1). The 18S primer was conjugated to a VIC fluorophore and utilized as an internal control for normalization. All qPCR assays were performed in triplicate and included a water control. A solution containing TaqMan Universal Master Mix (Thermo Fisher; #4369016), 18S primer, NIPSNAP1 primer and nuclease-free water was made for qPCR analysis. The prepared master mix was then combined with cDNA samples in a 96-well PCR plate (Thermo Scientific; #N8010560). The plates were then sealed using adhesive film (Thermo Scientific; #4360954) and centrifuged at 300 g for a total of five minutes at room temperature. After centrifugation, qPCR reactions were performed utilizing a QuantStudio Real-Time PCR System set to standard cycling conditions at a 20uL volume. Relative gene expression was calculated using the ΔΔCt method. ΔCt values were calculated as the difference in Ct values between the gene of interest and the 18S control (ΔCt = Ct_NIPSNAP1 – Ct_18s). ΔΔCt values were then calculated as the difference in ΔCt values between treatment groups (ΔΔCt = ΔCt_NIPSANP1 KO – ΔCt_control). Finaly, ΔΔCt values were transformed (2^-ΔΔCt_sample) and expressed as a percentage of lenti-control mRNA abundance.

### Intact Cell Respiration

To assess intact cell respiration, approximately 1–2×10^6 cells were harvested and centrifuged at 300 × g. After centrifugation, cells were then resuspended in either 0.5mL or 1mL Intact Cell Respiratory Media (bicarbonate free RPMI, pH set to 7.4 with HEPES and supplemented with 1% Penicillin/Streptomycin and 10% FBS) as previously described^42^. Viable cell count was then performed with Trypan Blue (0.4%) (Thermofisher; 15250–061). Basal respiration was then quantified, followed by FCCP titration (FC, 0.5–5μM). Antimycin A (Ant A, 0.5μM) was then added as a negative control to inhibit mitochondrial respiration.

### Cell Growth and Viability

Control (shScramble) and NIPSNAP KO HCT116 cells were seeded in a 24-well plate in triplicate at 0.2×10^6^ cells/mL in 1mL total volume per well of either RPMI-1640 (Thermo Scientific; 61870–036) or RPMI supplemented with 2% DSS. All media utilized was supplemented with 10% FBS and 1% penicillin/streptomycin. Once seeded, cells were collected for analysis at 24- and 48-hour time points. Wells were washed with PBS prior to lifting. Cells were then lifted off the plate using a 10-minute incubation of TrypLE (Gibco; 12604–013) at 37C. Once lifted, cells were neutralized using fresh RPMI and harvested for counting and viability assessment. To ensure all dead cells remained in the counts, we kept the PBS used for the initial wash and combined it with the harvested cells. Once harvested, the cells were assessed for growth and viability via Trypan Blue (0.4%) exclusion.

### Respiratory flux analysis in intact and permeabilized cells

High-resolution respirometry measurements were performed using the Oroboros Oxgraph-2k (O2k; Oroboros Instruments, Innsbruck, Austria) in a 1.0 mL reaction volume at 37°C. For each assay, 1–2×10^6 cells were harvested and centrifuged at 300 × g as previously described^42^. All cell data was normalized to live cell count, performed with Trypan Blue (0.4%). Cells were permeabilized by digitonin (15ug/mL) and exposed to the creatine kinase clamp technique as well as assessments for maximal respiratory capacity. The creatine kinase clamp technique was performed as previously described^19^ in permeabilized colon tissue. To assess maximal respiratory capacity, permeabilized cells were supplied with saturating amounts of carbon-based substrates and then exposed to the synthetic uncoupler FCCP. This separates the action of the electron transport system from that of ATP synthase, allowing the electron transport system to operate and respire at maximal capacity. For each assay, saturating amounts of carbon substrates and inhibitors were employed. Substrates and inhibitors utilized are indicated as follows: Creatine Kinase (CK − 20 U/mL), ATP (5 mM), PCR (5 mM, 14 mM, 20 mM), pyruvate (Pyr; 5 mM), malate (Mal; 1 mM), glutamate (Glut; 5 mM), Multi (Pyr; 5mM, Mal; 1mM, Glut; 5mM, Oct; 2mM, Succ; 5mM), octanoyl-carnitine (oct; 0.2 mM), succinate (Succ; 5 mM) cytochrome C (Cyt C, 10 μM), oligomycin (Oligo, 0.02 μM), rotenone (Rot, 0.5 μM), malonate (Malo, 20mM), antimycin A (Ant A, 0.5 μM),

### Statistical Analysis

Statistical analysis was performed using GraphPad Prism 10.2.2. Two-way ANOVA was utilized to analyze the effects of DSS on respiration. Three-way ANOVA was conducted to include NIPSNAP1 status in statistical comparisons. All data are represented as mean ± SEM and analysis were conducted with a significance level set at p < 0.05. Details of statistical analysis are included within figure legends. Figures were generated using Biorender and GraphPad Prism.

## Supplementary Material

Supplementary Files

This is a list of supplementary files associated with this preprint. Click to download.

- ColitisManuscriptSupplementaryTable1.xlsx
- ColitisManuscriptSupplementaryTable2.xlsx

## Data Availability

All data needed to evaluate the conclusions in the paper are present in the paper, with source data available in Supplementary Table 2. All raw data for proteomics experiments are available online using accession number “PXD065807” for Proteome Xchange40 and accession number “JPST003881” for jPOST Repository41. Proteomics data are also available in Supplementary Table 1.
